# Impact of the COVID-19 pandemic and control measures on type 2 diabetes care among South Asians: the perspective of healthcare providers in British Columbia, Canada

**DOI:** 10.1371/journal.pone.0359295

**Published:** 2026-10-23

**Authors:** Bushra Mahmood, Jia Hu, Tricia S. Tang, Prince Adu, Zaeema Naveed, Naveed Z. Janjua, Akshay Jain, Amandeep Nandra, Yasmin Banga, Arun Garg, Clara Iturra, Monica Garcha, Geoffrey McKee

**Affiliations:** 1 Division of Endocrinology, University of British Columbia, Vancouver, British Colombia, Canada; 2 BC Centre for Disease Control, Vancouver, British Colombia, Canada; 3 TLC Diabetes and Endocrinology, Surrey, British Colombia, Canada; 4 BC Cancer Agency, Vancouver, Canada; 5 Seattle Pacific University, Seattle, Washington, United States of America; 6 Fraser Health, Surrey, British Colombia, Canada; 7 Fraser Street Medical Clinic, Vancouver, British Colombia, Canada; 8 School of Population and Public Health, University of British Columbia, Vancouver, British Colombia, Canada; Touro University California College of Pharmacy, UNITED STATES OF AMERICA

## Abstract

**Background:**

British Columbia is one of the most ethnically diverse provinces in Canada, with South Asians emerging as one of the fastest-growing ethnic minority groups, burdened by a high incidence of type 2 diabetes. During the COVID-19 pandemic, many healthcare services shifted to virtual care to minimize infection transmission. Given the limited understanding of how primary care physicians in BC adapted their diabetes care approaches and the consequent impact on patient experience, this qualitative study aims to explore the effects of pandemic-related control measures on primary care diabetes services in BC from the perspectives of general practitioners, focusing particularly on their South Asian patients.

**Methods:**

We conducted semi-structured interviews with ten primary care physicians serving large South Asian patient populations. Interviews explored the pandemic’s effects on care delivery, provider adaptations, and consequences for South Asian patients with diabetes. Sessions were held in person or via Zoom/Teams and transcribed verbatim. Transcripts underwent thematic content analysis to identify recurring themes and insights regarding barriers, adaptations, and recommendations to improve diabetes care.

**Findings:**

The COVID-19 pandemic disrupted diabetes care, shifting services to virtual care and interrupting routine monitoring. This transition impacted specific aspect of diabetes care that can best be provided in-person (e.g., foot examinations, blood pressure checks and lifestyle counselling). The pandemic compounded existing issues, e.g., cost, concerns with side effects, and medication compliance among South Asian patients. Physicians also observed a general rise in anxiety and depression during the pandemic among their patients. Older South Asian patients faced additional cultural, language, and digital barriers; physicians struggled to obtain timely Hemoglobin A1c (HbA1c) tests. For many patients, a significant reliance on pharmacists embedded within certain physician’s clinics, ensured an uninterrupted access to diabetes medication and supplies.

**Conclusion:**

The COVID-19 pandemic disrupted diabetes care, with some groups facing additional cultural, language, and digital barriers. Our findings highlight the need for primary care reforms: improved telemedicine and EMR infrastructure, and policies to support virtual care. Bridging the digital divide and implementing targeted supports are essential to minimize care disruption for vulnerable populations during future pandemics or similar crises and ensure equity.

## Introduction

The COVID-19 pandemic significantly overwhelmed health systems world-wide and impacted individual patient care. Type 2 diabetes is one of the most common chronic conditions managed in primary care. In many countries public health control measures restricted access to routine diabetes care and changed care-seeking behaviour, which limited new diagnoses, testing, and management including access to medications. A global survey of health-care professionals from 47 countries reported that diabetes was the chronic condition most affected by COVID-19 due to disruptions in care [1]. On March 17, 2020, the British Columbia (BC) Provincial Health Officer declared a Public Health Emergency under the Emergency Program Act to support the province-wide response to the novel coronavirus pandemic [2]. Public health response measures included vaccination, physical distancing, wearing face masks, and restrictions on gathering sizes. These measures, while intended to minimize COVID-19-related illnesses and deaths, had substantial societal consequences [3]. As a result of these measures, compared to 2019−20, the rate of any primary care visits in BC declined in 2020−21, rebounding only slightly in 2021−22 [4]. To reduce COVID-19 transmission, many healthcare services transitioned to offering virtual care. Protocols were implemented quickly to deliver patient care by phone or video conference wherever possible [5]. Virtual healthcare expands access beyond distance and time, but cultural diversity, low health literacy, and limited English proficiency create barriers that require interpretation and care-navigation supports [6].

BC is one of the most ethnically diverse provinces in Canada with South Asians being one of the fastest growing ethnic minority groups representing 9.6% of the province population in 2021 [7]. South Asians are also particularly vulnerable to diabetes, with age-sex standardized prevalence rates for diabetes in BC being one of the second highest in the province – at 15.9% compared to 7.8% among Europeans [8]. Moreover, South Asians were also disproportionately impacted by the COVID-19 pandemic, with national mortality rates being the second highest at 31/100,000 population (after the Black population) compared to the national average of 22/100,000 [9]. This may have created more fear and anxiety among the South Asian community thus further impacting their health-seeking behaviors during the pandemic.

Due to limited knowledge about how primary care physicians in BC adapted their approach to diabetes care during the pandemic and the impact of these changes on patient experience, the primary objective of this qualitative study was to examine the effects of pandemic related control measures on primary care diabetes services in BC from the perspective of general practitioners (GPs) with a specific focus on their South Asian patients.

## Methods

### Recruitment

We conducted one-on-one, semi-structured interviews with primary care physicians in BC who reported significant representation (≥20%) of South Asian patients among their practices. Recruitment period lasted from June 1^st^, 2023 till November 1^st^, 2024. The majority of these physicians were recruited via the Vancouver Division of Family Practice, a not-for-profit society funded by the Government of BC and Doctors of BC that works in partnership with the BC Ministry of Health, Vancouver Coastal Health, Providence Health Care, and different community organizations. We initiated contact with the facilitator for the Membership Engagement and Collaboration program of the Division of the Vancouver Division of Family Practice via email. To support recruitment efforts, the Division featured information about the research in its biweekly e-newsletter. Subsequent participants were identified through snowball sampling and contacted via email, in-person meetings, or telephone. Interviews were conducted until data saturation was reached and no new themes or additional insights emerged from the interviews.

### Data collection

We created a semi-structured interview guide for this study (S1 File) to investigate the experiences of primary care physicians in providing diabetes care to their patients during the period when pandemic-related control measures were in place. The interview questions centered on the overall impact of COVID-19 on patients and healthcare providers, specifically regarding diabetes care. They explored how the pandemic affected care delivery and what changes, such as virtual or telephone visits, providers implemented. Additionally, the questions aimed to understand how these modifications influenced patient care- specifically for South Asian patients living with diabetes. Some of the interview questions were informed by emerging literature and therefore addressed themes that were identified a priori, including the impacts of the COVID‑19 pandemic on routine care and diabetes management, challenges associated with telemedicine, and the influence of lifestyle behaviors such as diet and physical activity. Some questions were developed based on COVID-19–related issues identified by members of our Clinical Advisory Panel specifically related to medication adherence. We did not collect any demographic information such as age or sex from our physician participants. The main reason was that our study focused on their professional perspectives and practice contexts rather than personal attributes. Collecting such data was not relevant to our research questions and could have increased the risk of deductive disclosure in a relatively small professional community. We also felt the physician community would not feel comfortable divulging this type of information. This approach was consistent with our ethics approval and ensured that interviews remained focused on physicians experiences of providing diabetes care during the pandemic.

All interviews were conducted between July 2023 and November 2024. Informed written consent of the healthcare providers was obtained at the beginning of each interview. Three out of the ten interviews were conducted in person at the physicians’ clinics, while the rest took place over Zoom (Zoom Communications, Inc CA, USA 2011) and Teams (Microsoft Teams 2017) virtual platforms. During the time of the interviews, no person except the interviewee was present in the room. Anticipating the profound impact of COVID-19 on mental and emotional health triggered by social isolation, loneliness, unresolved bereavement, grief and financial loss among our participants, we developed a list of resources across several categories, with a focus on immediate crisis intervention, culturally sensitive support, and specialized programs for those most impacted by the pandemic (S2 File).

All in-person interviews were recorded with a hand-held recorder. Remaining interviews were audio recorded using Zoom/Teams audio feature. Recruitment and analysis occurred concurrently, and as interviews progressed, recurring patterns became evident across participants. The final interviews did not generate substantially new codes or concepts, suggesting that sufficient thematic depth had been achieved for the study objectives.

This study was approved by University of British Columbia Behavioral Research Ethics Board (H20-02097). UBC ethics adheres to the principles outlined in the Declaration of Helsinki.

### Qualitative analysis

Four out of ten completed interviews were transcribed with the help of Otter.ai (CA, USA 2016, https://otter.ai) and re-checked manually for any inaccuracies. To protect participants, audio files were uploaded only for transcription, deleted immediately thereafter, and transcripts were stored on encrypted institutional servers. Audios did not have any names mentioned or any other identifiers. All transcripts were de‑identified before analysis.

The remaining six interviews were transcribed manually with quality checks of all interviews conducted by authors PA and BM. Interviews averaged 30 minutes in duration (range 15–45 minutes). Interviews were analyzed using **reflexive thematic analysis as described by Braun and Clarke** [10,11]. Two coders (BM and PA) independently read and coded the transcripts. Codes were reviewed iteratively through regular discussions of codes, categories, and emerging themes. Consistent with a reflexive thematic analysis approach, formal inter-coder reliability was not assessed; instead, analytical rigour was enhanced through peer debriefing, reflexive discussion, and collaborative review of interpretations.

Recruitment and analysis occurred concurrently, and data collection continued until no substantially new concepts or patterns were identified within the dataset.

Analysis proceeded through six iterative phases: familiarization with the data, generation of initial codes, searching for patterns across codes, reviewing candidate themes, defining and naming themes, and producing the final analytical narrative. Codes were initially generated inductively from the interview transcripts by PA and BM. Through discussions and iterative line by line review of coded excerpts, overlapping codes were merged, refined, or reorganized, resulting in a coding framework that captured recurring patterns across participants. Higher-order themes were subsequently developed by examining relationships among codes and identifying broader concepts that explained physicians’ experiences of diabetes care delivery among South Asian patients during the COVID-19 pandemic. To move beyond descriptive reporting, the analysis focused not only on what participants reported but also on the underlying meanings, contextual factors, and systemic challenges reflected in their accounts. Themes were interpreted in relation to the broader context of COVID-19–related healthcare disruptions, cultural and socioeconomic influences affecting South Asian populations, and physicians’ perspectives on barriers and adaptations in diabetes care. This interpretive process enabled the generation of analytic themes that explain how and why particular challenges emerged, rather than merely cataloguing reported experiences.

Research Team and Reflexivity: All interviews were conducted by the first author (BM) who is a female Research Fellow with extensive experience in moderating focus group discussions and conducting individual, one-on-one interviews. There was an existing relationship with at least three out of ten participants who agreed to be interviewed for this study.

Participants were also aware of the fact that the interviewer identified as a South Asian herself and was thus interested in researching the unique experiences of her community trying to access diabetes care during the COVID-19 pandemic. Although the interviewer was professionally acquainted with three participants, these relationships did not involve supervisory roles. The research team recognized that such familiarity could influence data collection by facilitating rapport and candid discussion, while also introducing the possibility of social desirability bias. To mitigate this, all participants were assured of confidentiality and reminded that the study aimed to understand their experiences rather than evaluate their clinical practice. Reflexivity was maintained throughout the research process through ongoing discussion among team members regarding how their professional backgrounds, sharing similar ethnic background and assumptions, and experiences might shape data collection, analysis, and interpretation. Coding and theme development were reviewed collaboratively to challenge individual assumptions and enhance the credibility and trustworthiness of the findings.

## Findings

We interviewed a total of ten physicians. Eight out of ten physicians served a large South Asian patient population, with proportions ranging from ≥20% to 95%. Two out of ten physicians had a South Asian population below 20%. A significant proportion of these patients were reportedly diagnosed with type 2 diabetes, highlighting the high burden of diabetes in this community. Physicians discussed the various challenges and issues they encountered while providing diabetes care during the pandemic. They also discussed how they modified care strategies to maintain support for patients living with diabetes, particularly those from South Asian communities. Below are major themes, some of which were identified a priori while others emerged from the interview data

1 **General impacts of the COVID-19 pandemic on routine care**

COVID-19 related measures reportedly had an immediate impact on the number of patients visiting their GPs. All physicians reported a significant reduction in in-person visits during the pandemic, with many consultations shifting to telemedicine. Some physicians noted that compared to their white patients, South Asian patients appeared to be more fearful of visiting clinics, hospitals, or labs as they were afraid of contracting the COVID-19, even when there was a better understanding of the transmission of the virus.

During the pandemic, telemedicine became a vital tool as many clinics transitioned to phone and video consultations to provide continuous care while minimizing the risk of COVID-19 transmission. However, in-person visits for certain aspects of diabetes management (e.g., foot examinations, blood pressure checks and lifestyle counselling) remained important and could not be performed via telemedicine.

Overall, physicians expressed satisfaction with the speed at which provincial government moved to shift health care delivery to virtual platforms, including full reimbursement for telemedicine visits. However, the majority of the physicians held the view that while telemedicine was advantageous, it could not and should not replace in-person care.

As one physician noted:

*“There’s some silver lining in this. I think having telemedicine has really significantly improved access to care. I just think that there should be a mandate that we [physicians] are required to do in-office visits. I think it’s ludicrous for people to think that they could just provide care only on virtual platforms.” Participant # 2*

Beyond general challenges with reduced in-person visits and the shift to telemedicine, South Asian patients often faced additional barriers due to language and cultural differences. For example, patients often struggled to identify medications by name, leading to confusion and potential errors. Family members often played a crucial role in facilitating telemedicine visits, especially for elderly patients who were not technologically proficient. According to one South Asian physician with a patient panel of whom almost 95% were of South Asian descent:

*“In in-person care, we are always asking the patient to bring their medications along when they come. We discuss each and every medication with the patient: How many of these pills do you take and at what time? We just verify. In virtual care, you can’t see when [what] the patient is taking. If we have to stop some medication we will say, ‘okay, stop this medication’. Patient will ask: ‘Oh - the round one?’ Well, then every pill is round – all pills are round. ‘The white one?’ Most of the pills are white. So this is the difficulty in virtual care... Most of the people are not educated. They have difficulty with English. So then this is another challenge - managing medication.” Participant # 7*

2 **Impacts related to diabetes screening, assessment and management:**
  a) **Impacts of decreased in-person visits:**

Physicians talked about the possible impact of reduced in-person visits on routine diabetes screening which may have resulted in delayed diagnosis of new cases. Additionally, disruptions in community-based screening programs such as those held at religious settings and at community health fairs may have affected early detection and management; however, one physician observed an uptick in screening among his relatively younger patient population primarily because a shift to virtual care which he believed provided them easy access to their family physician.

*“I believe because the access to care improved… younger people who have their day-to-day jobs, they didn’t have to physically come in to see me. So, I think more did end up having screened because it was easier for them to just follow up over the phone, they didn’t have to come in person, I think that was a barrier before where they actually had to come in-person to see me and some of them would have put off things…” Participant # 3*

Although telemedicine helped somewhat maintain continuity of care, it was reportedly not as effective as in-person visits for certain aspects of diabetes management, such as physical exam (blood pressure monitoring, monofilament tests), and comprehensive lifestyle counseling. Additionally, some physicians observed a possible decline in routine ophthalmological care, such as screening for retinopathy, mainly because patients may not perceive that as an imminent threat like heart attack or stroke. Routine screenings for these conditions, according to some physicians, probably took a back seat.

b) **Impacts of decreased lab access:**

Lab closures and reduced access to routine HbA1c screening reportedly led to decreased monitoring, less frequent check-ups, and delayed interventions. As noted by some physicians, these changes in diabetes care services could have resulted in poorer glycemic control and increased risk of complications. One physician did observe an increase in her patients’ mean HbA1c. Talking specifically about her South Asian patients, one physician observed the following:

*“I guess I also notice that in terms of continuous glucose monitoring, not a lot of my South Asian patients have a free style Libre or Dexcom. I know during the pandemic those who had those continuous glucose monitors could predict their A1Cs without having to go to the labs. So I guess that was less likely in the South Asian population.” Participant # 8*

While physicians believed that using home glucometers to monitor blood sugar levels was an important aspect of self-management, this could not be a sufficient substitute for other lab tests like the HbA1c.

c) **Impacts on medication adherence:**

Physicians provided insights on the various financial impacts of the pandemic on diabetes management, including loss of employment and income, the cost of medications, access to affordable food, and the financial strain on both patients and healthcare providers – all of which may also have impacted medication adherence and diabetes treatment plans. One participant talked about pandemic related lay-offs and the consequent loss of insurance coverage for medications that are expensive and may have been partly covered by employers insurance before. Additionally, since many clinics had reduced in-person visits, the health care providers were unable to give patients free medication samples like previously.

These factors all contributed to medication non-adherence – especially for South Asian patients. During the pandemic, assessing medication adherence via virtual/phone visits became even more challenging. As one physician noted:

*“South Asian patients need a lot more work than I would think non- South Asian. So we need to be on top of it: ‘take your medication, go for your blood tests, take your medication’. Right now, I feel I’m getting more face-to-face time - which is pushing them to go for blood tests and take their medications. Noncompliance is also slightly higher, I think in South Asian population.” Participant # 6.*

The pandemic compounded existing issues (e.g., cost, concerns with side effects, etc.) with medication compliance among South Asian patients.

*“And she was on Gliclazide, as well as Jardiance, and metformin. And she met with the clinical pharmacist, and for 14 months, she didn’t take Gliclazide or the Jardiance. Yeah, I was refilling them every time. And she didn’t take it because it was the cost and she didn’t want to be on multiple medications. So when her A1C wouldn’t be on target, I was either adding or whatever.” Participant # 6.*

3 **Impacts on patients living with diabetes**

Physicians provided their perspective on the impact of COVID-19 and its related control measures on their patients’ health seeking behaviors, difficulty with telemedicine, lifestyle habits, and mental health.

a) **Impact on health-seeking behaviors of patients:**

The COVID-19 pandemic had a profound impact on the health-seeking behaviors of patients with diabetes. For the fear of contracting COVID-19, many patients were reluctant to visit healthcare facilities resulting in delays in seeking care and routine check-ups. As mentioned earlier, South Asian patients seemed particularly fearful and avoided labs, even when their family doctor provided reassurances regarding its safety. They also put off attending diabetes clinics and group-based lifestyle counselling even when it was relatively safe to do so. As one physician observed:

*“ They [South Asian patients] would not go [for bloodwork] even when labs were okay - with social distance, they [still] wouldn’t go. I couldn’t get them to go to the lab. In summer, I would beg them like, you know, it’s not as cold, go, get at least one blood test done in summer for me. So, I had to literally push them to go for lab work…” Participant # 6.*

Physicians noted that most of their South Asian patients live in extended family arrangements, with multiple generations in the same household. The fear of infecting vulnerable elders likely further discouraged them from taking unnecessary risks by visiting healthcare facilities. As one physician observed:

*“I think there was more fear in that [South Asian] population. I don’t know if it was the patient or was it more the families [their parents] that they were concerned about…which led them a lot to maintain distance from clinics and hospitals. What was most concerning was when I would get phone calls from patients saying ‘you know, I had chest pain last week, and it was very bad, but I didn’t want to go to the hospital’ and I’m like, ‘What are you doing? You need to go to the hospital!’.” Participant # 5.*

Interestingly, despite COVID-19-related fear and anxiety and reluctance to leave their homes, physicians observed that South Asian patients were more willing to get vaccinated compared to Caucasian patients. This willingness was likely influenced by a paternalistic view of healthcare, strong community and family support, proactive measures by healthcare providers, and effective community outreach and education efforts from the provincial government. As one physician observed:

*“I think they [South Asians] are very good in that sense because immigrant populations- I am Latin American so a lot of Latin Americans- usually their parents live with them. We have multi-generational families living together. And for myself it was very important that my kids- we were all vaccinated to protect our parents and our grandparents. I find that it was also very important in South Asian families.”*

b) **Challenges of telemedicine:**

Rapid implementation of telemedicine during the pandemic may have posed some significant challenges for specific patient populations. Physicians noted that technological literacy, access to devices, and privacy/confidentiality concerns presented barriers for some groups. Some physicians articulated technological barriers faced by their South Asian patients including difficulties with audio/video calls, accessing emails, and using online platforms for healthcare.

*“I think the younger ones were fine…but I think the older patients, it was a little bit tough, because what would happen is even when phone calls would be set up by family members and then sometimes I would call, somebody would not pick up or if they picked up, they would just not be able to express that well, like especially people for whom English is a second language. I think it was tough for them. And booking an appointment was tough as well.” Participant # 3.*

Many of these patients did not have the necessary equipment, such as computers, smartphones, and especially printers, to facilitate virtual care. This made it difficult to receive and print lab requisitions or other important documents all of which may have differentially impacted their diabetes management. As one physician noted:

*“I think the biggest impact… that comes to my mind is the requisitions - how to print them. They [patients] would have to come to our office to pick them up because they didn’t have a printer at home. Or they’d have to contact their daughter or son or whatever to get support. I feel like that was the biggest barrier for me - not even to go to the lab, but like, how do I get this paper, and even faxing it to the main Life Labs was always an issue. They never got it. So I think that was probably the most frustrating thing – [how to] get blood work done. Because you didn’t have that piece of paper.” Participant # 3.*

c) **Lifestyle changes**

Lockdowns and restrictions on outdoor activities reportedly led to reduced physical activity. Some physicians noted that patients who were previously active, particularly regular gym-goers, struggled to maintain their exercise routines. Conversely, those who were not very active became even more sedentary. As one physician pointed out, since most South Asians are not regular gym-goers and prefer walking as their primary form of exercise, the closure of gyms and organized sports facilities had minimal impact on their overall physical activity levels.

Changes in dietary habits, including increased consumption of comfort foods and reduced access to healthy options were also common. Some physicians noted a weight increase in their patients once clinics re-opened. Moreover, several physicians raised the potential link between the high fat and high carbohydrate South Asian diet to worsening glycemic control. Although the recommendation to ‘ go and exercise and diet’ during the pandemic is important, this message needed to be ‘specific and individualized’ for the patient along the means and resources to do so.

One physician, who prioritizes preventative health, emphasized the importance of offering individualized care to patients. As a South Asian herself, she believed this gave her an advantage, allowing her to understand cultural nuances and provide culturally tailored advice on lifestyle behaviors to her patients:

*“ [lifestyle behaviors] can make a really big difference… even the tiniest things…When I say exercise to [patients], let’s say their back is hurting, which is a big thing for everybody. They think exercise means ‘let’s go for a run’. And I think that’s where we [physicians] have to flip the switch on them and say, it’s not about exercising in terms of running or jogging. It’s about stretching or doing core exercises or postural exercises. And I think we need to change the way we talk to patients…Yes, cardiovascular exercise is very, very important. But no one’s going to start running a marathon, right?” Participant # 2.*

Referral to Primary Care Networks (PCN) for free lifestyle counselling was a significant asset particularly during the pandemic. PCNs are community-based networks of health care professionals and clinics in some communities. Within these networks, family doctors and nurse practitioners work collaboratively with healthcare providers, including registered nurses, pharmacists, social workers, dieticians, mental health professionals, Indigenous and community providers and others.

d) **Mental health:**

The pandemic reportedly exacerbated mental health issues, including anxiety and depression, which in turn may have affected diabetes management. Social isolation further contributed to worsening mental health. Restrictions on social gatherings and community activities reduced the social support that many patients relied on, impacting their overall mental well-being and ability to manage their diabetes. As one physician noted:

*“ There is a very strong relation of mental health issues with diabetes - very, very strong relation. People with diabetes have mental health issues and people with mental health issues have diabetes – there is a very strong relationship. [This got worse during the pandemic] because of the isolation. Less social support. There’s no social support – you are just imprisoned in the home, you can’t get out. You can’t go to Mosque or Gurdwara. You can’t go to see someone. So the lack of social support was a big factor. And people’s mental health- it definitely was for sure affected.” Participant # 7.*

Several physicians observed a general rise in anxiety and depression during the pandemic among patients diagnosed with diabetes. However, it did not appear that patients were accessing care for these co-morbid mental health concern.

A few physicians noted that the South Asian population, in particular, experienced heightened fear and anxiety related to COVID-19, which impacted their care-seeking behavior. Some of this may have been attributed to misinformation, false theories, rumors and myths about COVID-19 that one physician believed they may have picked up either from their neighbors or community members in religious settings such as Gurdwaras. According to one physician, while younger South Asians were more forthcoming about mental health issues, older South Asians tended to keep to themselves, and mental health issues presented in other forms such self-medication with alcohol and other maladaptive behaviors.

4 **Adaptation strategies and enablers**

During the COVID-19 pandemic, physicians adapted their care strategies to ensure continuity of care for patients with diabetes. These adaptations highlight the flexibility and resilience of healthcare providers in ensuring continued patient care during the pandemic.

a) **Patient outreach via Electronic Medical Records (EMR) information:**

Many clinics proactively contacted high-risk patients to check on their well-being, remind them about medication refills, and ensure they were managing their health effectively. Utilizing EMRs to track patient health metrics and flag those needing follow-ups or missed tests helped providers stay on top of patient care. Some clinics used specific tools provided by their EMRs to generate links where a patient could then upload photos of medication list or skin lesions etc. Involving family members who were more comfortable with technology in virtual appointments and care discussions helped ensure that patients, especially the elderly South Asian patients, received the necessary support.

As one physician noted:

*“We reached out to patients at high risk for COVID and phoned them up to advise them to get vaccinated. We created an electronic medical record dashboard with anyone with chronic diseases such as heart failure, diabetes, COPD, MI stroke, and linked it to when medicines were running out. We would call them all one month before they ran out just to check in with them make sure that they knew about measures to reduce the acquisition of COVID.” Participant # 5.*

b) **The use of allied health – increasing pharmacy and chronic care coordinator collaboration:**

Several physicians also observed a significant reliance on pharmacists embedded within their clinics, with one physician reporting that, at one point, pharmacists became the only in-person contact for patients. Pharmacists ensured that patients’ medications were renewed without interruption, even if they were not able to come in for an appointment. These pharmacists handled medication refills and reviews, often acting as an additional set of ‘eyes and ears’ for the physicians. In clinics with a large South Asian patient population, some pharmacists could speak the patients’ native languages, which was particularly helpful. They also arranged for medication delivery services to ensure patients received their prescriptions without needing to visit the pharmacy. Additionally, some physicians mentioned an increased reliance on their clinic’s chronic care coordinators, most of whom could speak the same languages as the patients. These coordinators were responsible for reviewing charts of patients with chronic diseases, including diabetes, and proactively contacting high-risk patients to check on their well-being and provide advice on managing their health effectively.

c) **Community Enablers**

Physicians highlighted several community enablers that played a significant role in supporting diabetes care during the pandemic. These community enablers collectively helped mitigate the impact of the pandemic on diabetes care by providing accessible, culturally appropriate, and comprehensive support to patients. They played a vital role in ensuring continuity of care and helping patients manage their diabetes effectively despite the challenges posed by the pandemic.

According to some physicians, having **a dedicated clinical pharmacist on their team** was very helpful during the pandemic as these pharmacists continued to provide medication reviews and counseling, especially for patients who had difficulty understanding medication changes.

As one physician observed:

*“So we also have pharmacists, in our clinic - clinic pharmacists that see patients. And typically, those are the diabetic patients, because they’re always on multiple medications. And so they do med[ication] reviews, which are- it’s just such a great service to have. So they’re like a second eyes and ears on our patients.” Participant # 2.*

Dietitians and nutrition programs were crucial for providing dietary advice tailored to cultural preferences. Some physicians mentioned programs like the Surrey Diabetes Education Program in Surrey (the second most populated city in BC with one of the largest South Asian populations in North America) offered education in languages like Punjabi, which helped patients understand and manage their diets better. PCN in certain communities continued to provide a range of services, including access to dietitians, counselors, and social workers. These networks, according to these physicians, helped in offering comprehensive care and support to their patients, especially those with language barriers or financial constraints. Another physician talked about programs like “Be Well” in Burnaby, (third largest city in BC), which focused on behavior change and was adapted to virtual delivery, provided support through health coaches, kinesiologists, and dietitians. These programs, according to this physician, helped in increasing physical activity and reducing stress and depression scores among patients.

Physicians noted that South Asian places of worship—such as gurdwaras, temples, and mosques—played a vital role in sharing information about COVID‑19 and diabetes management They provided a trusted source of support and helped in organizing vaccination drives and health education sessions. One of the physicians mentioned how the South Asian Health Institute based within the Fraser Health Authority went to gurdwaras to provide information about COVID-19 and set up vaccination clinics, which helped in dispelling fears and encouraging people to seek medical care.

Additional illustrative quotes linked with these major themes and sub-themes are captured in Table 1.

**Table 1:** Physicians perspective about providing diabetes care for South Asian patients during COVID-19 pandemic in BC, Canada.

Themes	Illustrative quotes from primary care physicians
Immediate impact on diabetes care	*“ Yeah, that first year, I think was really challenging- the 2020 year… I think we were all scrambling to figure out how can we communicate with patients, and people not getting their blood work, not going out…”*
	*“ Medications renewals, we were doing – we were not declining for any patients existing medications renewal. But lab follow up, we were not having – it was kind of little bit blind process because they have the three month HbA1Cs, which most diabetic patients do regularly. They didn’t have the opportunity to do that.”*
	*“ All patients were very adversely affected. We were providing care virtually. Physical care is always the best. Virtual is virtual- yeah-it is not as perfect as physical assessment.”*
	*“ So during the time when it was, you know, heightened, definitely during those times, if their A1C, let’s say, in the last six months, or even prior to was relatively stable, we didn’t sort of push on getting it every three months, you know, like, kind of stretching it to six months… So we kind of worked around that, but I don’t, I mean, there’s probably a bit of a delay, but I still think we were able to provide care, and they were still seeking care whether it was mainly then on the phone, I would say the thing that lacked the most was that we weren’t able to do physical exams, like the monofilament test, you know, things like that, maybe checking their blood pressure in the office…”*
Specific challenges for South Asian patients	*“...For South Asian population, the challenge is that they -a lot of them are not educated. Right? And so, and I don’t think it’s that they don’t always know – whether it’s about, you know, an [education] thing or not, I think they just don’t want to try. Right? They’re just like, ‘oh, I don’t know, like, you know, ask so and so.’ And so, you know, I would explain to them or get them to sort of, again, I think myself being South Asian, and knowing how South Asians function, and having to deal with it myself on a day to day basis in my own family, has really been able to help me in terms of helping with my South Asian [patient] population.”*
	*“I think the younger ones were fine, like you know, but I think the older patients, it was a little bit tough, because what would happen is even phone calls, you know would be set up by family members. And then sometimes I would call, somebody would not pick up or if they picked up, you know, they would just not be able to express that well, like especially people for whom English is a second language, right. I think though it was tough for them. And booking an appointment was tough as well.”*
	*“Lot of them- especially the elderly South Asians, definitely, because a lot of them would be foot traffic coming to the office. Most of the time for them if they had a family supporting them, it was okay. But if they had to call or they had to organize their appointments, I think it probably did [adversely impact]. And again, they were terrified to come to the office, I guess right?... I could not like even get a telephone appointment with a lot of them.”*
	*“ The South Asian patients, especially the seniors, mostly they have issues with the language. But we have the staff, which is also South Asian, so they have no difficulty in fixing them the appointments… they just pick up the phone. Logging into the computers for the South Asian community is a big problem. Very few percentages of people are familiar with computer [technology] you know. So most of the elderly, they are, they just pick up phone. And they speak in the language and tell what they want. If they have any computer issues they have help of their grandchildren.”*
Adaptation strategies and enablers	*“ We have chronic disease... coordinators in our clinic, a couple of people that work with us who go through all [patients] files, and we call people if they haven’t had their bloodwork done, or they if they haven’t, if their A1C was not on target or they’re missing urine ACR, or they’re missing the LDL targets.”*
	*“ So I think that’s one of the things that I realized that most of our patients were working with a small pharmacies or even and they got more hands on care, I think so that made it easier.”*
	*“ Well, the first thing is that during the pandemic, the enablers of self-management still stem from system changes such as telemedicine and they [government] should have also supported clinicians in providing them electronic medical record mining staff people that could have helped them to identify patients at risk, or who needed more support. They should have provided more telephone services and support such as translation services or personal trainers or pharmacist that could have done med reviews. For example, we had a medication review program where we had a Punjabi pharmacist able to translate and explain everything to patients. And if the government was really smart, they would have been able to extend from in office visits to telemedicine visits and we would have been able to maintain that program. But very short sighted they were trying to save money in a medication review program…”*
	*“ I think having the frequent weekly information from public health advocate, ‘this is what COVID is, this is what we know, who gets prioritized in terms of getting their COVID vaccines’ was helpful, there were certain categories, clinically, extremely vulnerable. So I think that kind of helped. If you’re seeing most patients with diabetes, maybe some of them are more in control, [some not] and getting those prioritized, because I think they retrospectively said diabetes was an independent risk factor [for COVID-19].”*
Shift to telemedicine	*“ And completely transitioning to virtual care was a little bit challenging initially, I think phone calls did help... And if the payment model had not adapted, using video would have been very challenging…”*
	*“ I had a patient actually…and over the phone, it was fine. We talked all the time, but I saw her in person and she must have lost 50, 60 pounds. And had I not seen her in person I think I wouldn’t have understood the magnitude of how [much weight she had lost] when she said … I’m losing a little [weight]. So that was really shocking to me to see or even patients who are diabetic that have gained weight, you know, so seeing them in person after so long, so many people had gained weight…you can’t judge that even on video call, right? Because angles make a big difference. So yes, I definitely feel like in-person [visit] is very important still at least once a year we should be seeing patients for diabetes care. Beyond that I don’t think it’s all that important. You can manage diabetes without having to physically see them every time. It’s a lot of bunch of numbers and targets that you’re reaching and just checking in to make sure if there’s compliance and all that kind of stuff.”*
	*“ I think just the pandemic did help us, you know, bring care to like the 21st century, essentially, I think, you know, having patients come in for some things that can be delivered via virtually…So I think being able to deliver care virtually, and being able to see them in person has, I think will improve outcomes and it’s helping us actually identify more diabetics and treat them at an earlier stage and get them more involved in their care. Right? Before it was so much work to come in. If I was a diabetic and having to go see my doctor three times a year… in person would be a challenge. It would be very challenging to do that because that means you’re compromising something else. And… if you are feeling good, people do compromise on their care… and they just think that ‘well I’m feeling okay, I’ll be fine’. Right? But just having access to virtual or in- person, if you want has changed your care and I think it’s going to improve outcomes too…”*
Lifestyle changes	*“ Yeah, I’m big on lifestyle changes. I feel like it can go a long way by making small little changes. And I think that’s where we have to, as healthcare providers not be like, ‘Okay, eat healthy and exercise’. Like, that’s not what it’s about. It’s like saying, ‘Okay…what can you do? Can you go for a walk for 10 minutes, can you and I think, I wish we had a little bit more support with that in our community, because I feel like, that’s where a lot of them can improve. I mean, we can keep adding medications over and over and over and over again. But I think in the South Asian population, having education on food, and diet is huge.”*
	*“ It [prescribed exercise] has to be specific for what we’re telling them[patients] for what they need to do. Yes, cardiovascular exercise is very, very important. But no one’s going to start running a marathon, right? I think that when they think ‘exercise’ or thinking about those people at the gym, and like running on the treadmill, and they’re like, ‘Yeah, I’m not going to do that in my suit and salwar [baggy pants]’, right…Everything needs to be individualized…we are not cookie cutter doctors, right? …We look at the patient, we decide like what is it that you can do differently? So we need to take that time to discuss that with patients. And I wish we had more time, which now with the LFP [longitudinal family physician payment model] model, it has been a lot better.”*

## Discussion

The COVID-19 pandemic significantly impacted diabetes care, with a shift to virtual care and subsequent disruptions in monitoring and management. South Asian patients, particularly those who were older, faced additional obstacles due to cultural, language, and technological barriers. However, proactive measures by physicians, community support, and culturally relevant education reportedly helped to mitigate some of these issues.

The impact of reduced in-person visits and increased telemedicine led to a reduction in visits and significantly hampered the ability to delivery physical exams and other aspects of care requiring an in-person component (e.g., lab testing). Telemedicine allowed for some continuity of care and was convenient for some patients but had its challenges. In the context of diabetes care, future research is needed to improve processes for the ability to obtain laboratory tests like HbA1C and reliable measures of weight and blood pressure during shifts to telemedicine [12] – specifically among South Asian patients where, according to the physicians observations, use of continuous glucose monitors and home-based blood pressure monitors is sub-optimal. Moreover, the digital divide across various vulnerable population groups needs to be acknowledged. Physicians noted difficulties experienced by their South Asian patients, particularly the elderly, in scheduling and maintaining virtual or phone appointments. Proficiency in reading and writing in English is often assumed to be a prerequisite for the effective utilization of digital health services [13]. In a study examining the use of digital health among senior immigrants in Canada, only 25% of Punjabi participants reported having advanced English proficiency [14]. Other studies have highlighted similar issues, such as the lack of internet and phone access, challenges faced by older adults with limited technological skills who prefer traditional methods [15,16], and racial disparities in telemedicine usage, with minority groups using these services less frequently than White individuals [17,18].

Physicians also reported that the pandemic led to significant impacts on patient behaviours. There were significant changes in the health-seeking behaviours of patients, particularly among the South Asian community who were particularly fearful of COVID-19. Additionally, there were significant impacts on patient lifestyle, including reduced levels of physical activity, changes in diet, and loss of employment which contributed to reduced income and insurance to pay for medications. Significant negative impacts on physical activity and diet have also been observed by other population level studies across the globe. A systematic review analyzing the impact of COVID-19 pandemic on lifestyles and mental health concluded an uptick in unhealthy lifestyle patterns encompassing reduced physical activity, increased sedentary behavior, augmented screen time, disturbed work and sleep schedules, increased smoking and alcohol consumption, and mental health disorders, such as anxiety and depression [19]. A further reduction of physical activity in South Asians – a sub-group already classified as highly sedentary and lowest levels of physical activity when compared with other ethnic groups [20,21] is of concern and may have far reaching implications.

The family physicians in our study observed a surge in mental health concerns in their diabetes patients stemming from fear of the unknown, financial insecurity, social isolation and a constant stress of infecting their loved ones. According to a WHO report, the COVID-19 pandemic triggered a 25% global increase in anxiety and depression, creating a lasting mental health crisis [22]. Furthermore, evidence suggests a bidirectional relationship between diabetes and depression [23,24]. Studies show a significant increase in prevalence of depression symptoms, generalized anxiety symptoms and distress, as well as significantly decreased health status in diabetes patients after the initial COVID-19 outbreak [25]. According to the Survey on COVID-19 and mental Health, in Canada, by the end of the pandemic, more than 8 out of 10 people reported experiencing negative impacts from the COVID-19 pandemic [26]. These studies thus reaffirm what we heard in our interviews and highlight the need to have specific support systems for patients living with diabetes in pandemic-like situations.

Apart from the transition to telemedicine, physicians spoke of several other adaptations that supported quality care for their patients. Physicians leveraged their EMRs to optimize patient outreach and to identify higher-risk patients. Additionally, allied health was fulsomely engaged to support patient care. and played an important supporting role. For example, pharmacists took on added responsibilities to ensure continuity of medications and review of medical charts to flag patients at high risk of hyperglycemia and related complications. Pharmacy practice models have highlighted community pharmacies’ unique position in connecting individuals to care [27,28] and the many contributions community-based pharmaceutical services provide in reducing racial and ethnic healthcare disparities [28]. In the context of diabetes care, these models of care hold promise for marginalized populations that are known to have higher blood glucose and higher rates of diabetes-related complications [29,30]. Community organizations also played a role in educating and supporting patients with both COVID-19 and diabetes.

### Strengths and Limitations

To our knowledge, this is the first research study in BC exploring the perspectives of primary care physicians delivering care for type 2 diabetes during the COVID-19 pandemic with a specific focus on the South Asian population. This research highlights some unique challenges that both physicians and patients from this community experienced while delivering or receiving care for their diabetes. Most of the physicians we interviewed were also South Asian and they were able to offer a more insightful perspective on the challenges their South Asian patients may have faced when seeking diabetes care.

Our study has some limitations. Our study sample was recruited via snowball sampling that which thus may have introduced selection bias [31].,. Snowball sampling was used to identify eligible physicians with relevant clinical experience; however, this strategy may have resulted in participants being recruited from similar professional networks, potentially limiting the range of perspectives captured and the generalizability of our findings. This limitation has been considered when interpreting the findings.

We only interviewed primary care physicians and no allied health providers (e.g., nurses, nurse practitioners, pharmacists). Additionally, we did not interview patients directly, so our conclusions are based on what their physicians shared. Consequently, the findings reflect physicians’ perceptions and interpretations of South Asian patients’ experiences rather than patient-reported experiences themselves. Provider accounts may be influenced by clinical observations, assumptions, or biases and should therefore be interpreted accordingly. Patient interviews were conducted as part of a separate study; however, the decision was made to analyze and report these data independently to ensure sufficient depth of analysis and fair representation of both participant groups. Including both physician and patient datasets within a single manuscript would have risked oversimplifying the experiences of either population.

Furthermore, this study was conducted using an inductive qualitative approach and was not guided by a pre-specified theoretical or conceptual framework. While this allowed themes to emerge directly from participants’ accounts, the absence of an explicit framework may have limited the depth of theoretical interpretation. Frameworks related to cultural competence, health behaviours, communication, or the social determinants of health may have provided additional insight into the findings. Future qualitative studies in this area should consider incorporating an appropriate theoretical framework during study design to strengthen analytical depth and interpretation.

We acknowledge that the interviewer’s existing professional relationship with three participants may have influenced the interview dynamics. This relationship could have facilitated trust and openness but may also have introduced response bias or shaped interpretation. We mitigated this by applying consistent interview protocols, reminding the interviewees that the major purpose of the study was only to capture their experiences and not an evaluation of their clinical practice, triangulating across multiple participants, and engaging in reflexive discussions during analysis. Lastly, due to recruitment challenges, it took longer than expected to complete all the interviews. Our findings thus may be potentially limited by physicians’ recall of events during the pandemic.

## Conclusion

The COVID-19 pandemic significantly impacted diabetes care, with some populations experiencing additional challenges due to cultural, language, and technological barriers. Our findings highlight the resilience of primary care physicians, evident in their ability to adapt, innovate, collaborate, and advocate for their patients, ensuring that high-quality care was maintained even in the face of unprecedented challenges. The impact of the pandemic on primary care practices establishes the need for continued primary care reforms including enhancements in infrastructure for telemedicine and EMRs, and introduction of policies and mechanisms, such as new billing codes, to support the shift to virtual care. Addressing the digital divide by ensuring that all patients, especially marginalized groups, older populations, and patients identifying as a visible minority have access to the necessary technology and internet services is imperative for health equity. Additionally, fostering collaboration between different healthcare providers, such as physicians, pharmacists, and community health workers is important to ensure comprehensive care. This may include creating integrated care models and communication platforms. Furthermore, recognizing and addressing the mental health impacts of pandemics by integrating mental health services into primary care is essential. Finally, in the context of future pandemics, it is crucial to establish policies that enable flexible and adaptive healthcare delivery models. This involves making regulatory adjustments to facilitate the swift implementation of virtual care and other innovative solutions, ensuring that healthcare systems can quickly adapt to changing circumstances and continue providing essential services.

## Supporting information

S1 File Interview Guide. (PDF)

S2 File Resources for Mental Health. (PDF)
