# Supplementary material for: Impact of the COVID-19 pandemic and control measures on type 2 diabetes care among South Asians: the perspective of healthcare providers in British Columbia, Canada
Source: PLoS One. 2026 Oct 23;21(10):e0359295. doi: 10.1371/journal.pone.0359295 (PMC13693937; doi:10.1371/journal.pone.0359295)
Supplement: Supplementary file 1 [file pone.0359295.s001.pdf]

Question Guide for Primary Care physicians

[Note: subject to change]

Thank you for your time.

In March of 2020, the government of BC began to implement control measures for COVID-19, many of which lasted for a considerable portion of the pandemic. Some of these measures like physical distancing requirements, mandatory quarantines, reallocation of healthcare staff, drastic reductions in patient-provider appointments, and/or replacement of in-person appointments with virtual appointments may have caused disruptions in services for patients with chronic illnesses, including diabetes care.

The purpose of this short interview is to get your perspective on the impact of the pandemic and public health measures on routine diabetes care to your patients.

Context

1. What proportion of your patient population would identify as South Asian?
2. What proportion of your South Asian patient population is living with type 2 diabetes – can you give us a rough estimate?
3. How many patients living with diabetes did you normally see in a week/month pre-pandemic?
    a. Did you see a reduction in this number during the pandemic?
    b. How large was this reduction?
    c. How long did it last?
    d. Did you notice any specific changes for your South Asian patients living with diabetes?

Impacts on diabetes care

4. How do you believe effective diabetes care within your practice may have been impacted by the pandemic and control measures? (Physical distancing, fear of infection)
    a. Were there any specific impacts on South Asian patients?

Prompts (not exhaustive)

- Screening
- Routine tests
- Management of co-morbidities
- Management of complications

- Access to allied services – dieticians etc.

5. How did you modify your delivery of care during the pandemic (Ex. Virtual, telephone, restricted walk-in appointments)?
    a. What type of feedback , if any, did you receive on these modifications from your South Asian patients?

6. Were there specific public health measures that you feel had the greatest impact?
    a. Were there any specific impacts on South Asian patients?

7. How do you believe concerns about COVID-19 infection impact care-seeking behaviour among patients living with diabetes?
    a. Were there any specific impacts on South Asian patients?

Experience and Self-management of diabetes

8. What challenges, if any, did your patients describe with managing their diabetes during the pandemic?
   Prompts:
    a. Experiencing diabetes complications
    b. Experiencing co-morbidities
    c. Mental health issues
    d. Severe COVID-19
    e. Lifestyle measures (physical activity, nutrition etc.)
    f. Glycemic monitoring and control
    g. Medication adherence

How about your South Asian patients?

Did you prescribe lifestyle changes (diet and PA) to your patients during the pandemic?

Enablers for diabetes care and management

9. Are there any characteristics of your practice that you feel enabled care for diabetes during the pandemic?

   Among South Asian patients?

10. Are there any best practices or community enablers that you feel enabled better self-management of diabetes among patients in your practice?
    a. Among South Asian patients?
        i. Community support groups
        ii. Non-governmental organizations
        iii. Gurdwara/Temple/Mosque based groups
