# Supplementary material for: Impact of the COVID-19 pandemic and control measures on type 2 diabetes care among South Asians: the perspective of healthcare providers in British Columbia, Canada
Source: PLoS One. 2026 Oct 23;21(10):e0359295. doi: 10.1371/journal.pone.0359295 (PMC13693937; doi:10.1371/journal.pone.0359295)
Supplement: Supplementary file 2 [file pone.0359295.s002.pdf]

# Mental Health Resources for South Asians

**South Asian Health Institute Mental Health Resources:**

https://www.fraserhealth.ca/health-topics-a-to-z/mental-health-and-substance-use

Mental wellness: https://www.fraserhealth.ca/health-topics-a-to-z/mental-health-and-substance-use/mental-wellness

Mental health resources to support you during COVID-19: https://www.fraserhealth.ca/health-topics-a-to-z/mental-health-and-substance-use/mental-health-resources-to-support-you-during-covid-19

**South Asian Mental Health Alliance:**

http://samhaa.org/

For Mental Health Professionals / Agencies

Please contact Raman Khaira: ramankhaira@samhaa.org

**HealthLink BC**: Mental Health and COVID-19 (Resources available in Punjabi and Hindi)

https://www.healthlinkbc.ca/more/health-features/coronavirus-disease-covid-19/mental-health-covid

**BCCDC:** Mental well-being during COVID-19: Find links and resources to help with mental well-being during COVID-19.

http://www.bccdc.ca/health-info/diseases-conditions/covid-19/about-covid-19/mental-well-being-during-covid-19

- Free or low-cost counselling is available virtually, such as through Wellness Together Canada. Courses and programs to support mental well-being are also available online.
- The Canadian Mental Health Association has COVID-19 specific mental health information and supports.

- They offer a free self-assessment that helps you reflect on your overall well-being. It helps to find the resources most useful to you.
- Provides free programs such as BounceBack®, designed to help manage low mood, mild to moderate depression, anxiety, stress or worry.
